# Hospital Meetings, &C.

**Published:** 1898-04-02

**Authors:** 


### HOSPITAL MEETINGS, &C.

### LONDON HOMŒOPATHIC HOSPITAL.

Mr. J. P. Stilwell (chairman of the Board of Management), who presided at the annual meeting of the London Homœopathic Hospital, held at the board room on Thursday, the 24th ult., in moving the adoption of the report, referred to the losses sustained by the hospital in the deaths of the Duchess of Teck and Mr. Hugh Cameron, the latter of whom had been associated with the hospital since its inception forty-eight years ago. Continuing, he said that one of the most striking things in the report was the increase in the number of patients treated during 1897. The in-patients numbered 1,064 as compared with 1,031 in 1896, and the out-patients 16,899 as compared with 14,514. This, he thought, was the result, in the first place, of the appreciation of the work carried on there by the staff; and, in the second place, to the fact that homœopathy was found by the poor to be very much more helpful to them than any other system of medicine. They found that the period of sickness was shortened, that the recovery was quicker, and that their strength was not undermined by treatment which in other institutions was called "heroic." Post-graduate lectures, he said, had been lately introduced, and the medical staff had arranged to carry on during the summer session daily lectures and demonstrations embracing general medicine and surgery and other special branches. This, he hoped, would prove a great help to homœopathy. He also referred to the fact that the whole amount—£48,000 odd—needed for rebuilding the hospital had been raised, and there was a small balance over—£340—which had been paid in to the general fund. In order to provide for the working of the hospital in its present enlarged state, further annual subscriptions, amounting to about £2,000, would be required.

The report was adopted.

Captain Cundy, in proposing that a sum of £2,000 be taken from the invested funds of the hospital for the purpose of paying off the balance of the cost of rebuilding and furnishing the nurses' home, also referred to the post-graduate lectures given at the hospital, saying that it was necessary, if they were to keep filling up the ranks of homœopaths as they were thinned by death, to have a school where medical recruits could be trained. That was why they had inaugurated these lectures.

Miss J. Durning Smith seconded, and the resolution was agreed to.

The president, vice-presidents, board of management, &c., were re-elected, and votes of thanks to the officers and the chairman concluded the proceedings.

---

### BRITISH LYING-IN HOSPITAL ANNUAL MEETING.

The annual meeting of this, the oldest charity of this kind in London, took place at the hospital in Endell Street, W.C., on March 24th, at five p.m. Considerable changes have occurred amongst the officers during the year. Mr. W. S. Knight, the secretary, died after a very short tenure of office, and Miss Freeman, the valued matron of the hospital for the past thirty years, has resigned in order to enjoy her well-earned liberty. Mr. A. C. Wickins has been elected secretary, and Miss Cook matron. The committee have plans on foot for reconstructing and renovating the building on a considerable scale, and thus bringing it in line with modern requirements. The nurses' sleeping accommodation will be greatly improved. The number of in-patients was 187, and of out-patients 390, making a total of 577. Only one, and she was admitted in a dying condition, succumbed. It is not sufficiently well known that only married women are admitted to the benefits of this charity, and that very comfortable arrangements are made for paying patients at the modest sum of £2 2s. a week, inclusive even of medical attendance if necessary, the matron herself attending such cases. This is undeniably a boon to poorer gentlefolk, who are enabled by its means to leave all domestic worries, and enjoy a quiet time at less expense than at home. Most of the modern conveniences are in use in spite of the antiquated and inconvenient building. Dr. Flux, of 5, Burlington Street, has been appointed anæsthetist; incubators are in use in every ward (this for the instruction of the pupils more than for the necessities of the infants); electric light, glass and metal instrument case, &c.; the improvements most needed being on the side of physical comfort, especially for the nurses. Mr. Charles E. Farmer took the chair, and moved the adoption of the report, and Mr. Algernon Strickland seconded it. There were also present Mr. Horace F. Cox, and the Rev. Prebendary Henry W. Parry-Richards. There is a balance of £33 in hand after investing £403, the total income having been £2,030.

---

### NORTH LONDON HOSPITAL FOR CONSUMPTION.

Mr. F. C. Mocatta presided at the annual Court of Governors of this hospital, held at 41, Fitzroy Square, on Wednesday, March 23rd. There were also present, among others, the Rev. S. B. Burnaby, Mr. B. A. Lyon, and Mr. W. J. Malcolm. The Chairman, in dealing with the report, commented on the fact that the usefulness of the institution was interfered with in consequence of the want of funds. There was room in the hospital for twenty more beds, but until more financial aid was forthcoming the accommodation could not be utilised. In order to make use of the hospital to the full an annual increase of £2,500 is required, a sum the committee are endeavouring to raise in connection with the festival dinner to be held on May 19th, when H.R.H. the Duke of Cambridge will preside. During 1897 the total income, including £1,500 from the Hampstead Diamond Jubilee Fund, and £262 from the Prince of Wales's Hospital Fund, amounted to £5,158, and the total expenditure to £5,253, thus leaving a balance of £95 on the wrong side. The committee state that, notwithstanding the issue of a strong appeal, the falling-off in receipts made it necessary for them to borrow a further £500, raising their indebtedness to their banker's to £1,000. Unpaid accounts at December 31st amounted to £1,127. The mortgages on the buildings, they add, remain the same, and the interest on these continue to be a serious drain on the income of the charity. By careful supervision of expenditure, a reduction under nearly every heading has, the report states, been effected as compared with 1896. The cost per bed, which was £106 3s. 6d. in 1894, £96 10s. 6d. in 1895, and £86 18s. in 1896, was in 1897 reduced further to £82. Coming to the work performed by the hospital during the year, 430 in-patients were treated, and, on the recommendation of the physicians, 121 were granted three weeks' extension of stay in the institution. The death-rate was only 4·6 per cent., as against 5·4 in 1896, and the majority of the patients left the hospital greatly improved in health. The out-patients numbered 3,279, of whom 858 attended at Hampstead and the others at Fitzroy Square. To Dr. Forbes Winslow, who has retired from the position of honorary physician, and to Miss Elphick, who has resigned the position of matron to take up another appointment, the committee express warm thanks for their services to the hospital. The success achieved by the institution is, they declare, largely due to Miss Elphick's capacity.

---

### ROYAL EYE HOSPITAL.

At the annual meeting of the Royal Eye Hospital, held on Wednesday, the 23rd ult., there were three governors present. Professor Malcolm McHardy took the chair, and stated that he held a number of proxies so that the meeting was properly constituted under the rules. The report, which was adopted, stated that during the year 1897 405 in-patients and 15,038 new out-patients had been treated. There were two empty wards containing 14 beds which could not be opened owing to lack of funds. The ordinary income had amounted to £1,656, receipts from the Jubilee seats to £1,134, and from legacies to £709. The ordinary expenditure was £2,824, and the extraordinary expenditure on repairs and building improvements £257. It is not possible from the published accounts to tell what were the expenses incurred in obtaining the £1,134 from seats to view the Jubilee procession, nor is it stated whether this £1,134 is the net or the gross proceeds from this source.

